# A systematic review and network meta-analysis protocol of neoadjuvant treatments for patients with gastric cancer

**DOI:** 10.1097/MD.0000000000010392

**Published:** 2018-04-13

**Authors:** Bo Long, Ze-yuan Yu, Qiong Li, Heng-rui Du, Zhen-jiang Wang, Hao Zhan, Zuo-yi Jiao

**Affiliations:** a Department of First General Surgery, Lanzhou University Second Hospital; b Department of Endocrinology, Lanzhou University First Hospital, Lanzhou, China.

**Keywords:** 5-year survival rates, Bayesian, gastric cancer, neoadjuvant treatments, perioperative mortalities, protocol, surgery, survival time, total mortalities

## Abstract

**Background:**

National Comprehensive Cancer Network (NCCN) guidelines recommend surgery, chemotherapy, and radiation therapy for gastric cancer patients. Neoadjuvant treatments as the administration of therapeutic agents before a main treatment gained in more and more attention. However, the role of neoadjuvant treatments is still controversial. The main aim of this systematic review and network meta-analysis is to assess the relative efficacy of different neoadjuvant treatment regimens for gastric cancer using network meta-analysis method.

**Methods:**

We will search 5 electronic databases to identify randomized controlled trials (RCTs) and non-RCTs compared the efficacy differences of surgery alone (S), preoperative chemotherapy follow by surgery (CTS), preoperative radiotherapy follow by surgery (RTS), and preoperative chemoradiotherapy follow by surgery (CRTS) for patients with gastric cancer. The risk of bias tool from the Cochrane Handbook version 5.1.0 will be used to assess the risk of bias of RCTs, and the risk of bias in nonrandomized studies of interventions (ROBINS-I) for non-RCTs. Data will be analyzed using R-3.4.1 software.

**Results and conclusion:**

The results of present network meta-analysis will estimate the relative efficacy among all interventions and rank the interventions even if head-to-head comparisons are lacking and will provide more evidence for clinicians, researchers, and patients in the management of gastric cancer.

Protocol registration number: CRD42017074956

## 1 Introduction

Gastric cancer (GC) is the fourth most prevalent malignancies and the second leading cause of cancer death worldwide, and the incidence of GC is relatively high in Asian, especially in China, although the incidence and the cancer-related mortality have been steadily decreasing during the past century.^[1–3]^ The main reason for the decline of the incidence of GC perhaps is advances in screening, surgery, the use of chemotherapy and radiation measures in treatment regimens.^[4]^ National Comprehensive Cancer Network (NCCN) guidelines also recommend surgery, chemotherapy, and radiation therapy for GC patients.^[5]^ Surgery is the standard treatment for GC patients. After curative resection, 5-year survival rate of early stage GC is up to 90%.^[4]^ However, the prognosis of GC patients is mostly determined by the stage of the disease, and the majority of the GC patients are diagnosed as advanced GC at the time of initial presentation, for the advanced GC patients the 5-year survival rate is only 10% to 30% even after curative resection.^[6]^

Neoadjuvant treatments are the administration of therapeutic agents before a main treatment. In 1989, the first report of neoadjuvant treatments in treatment of GC patients has been published and gained increasing attention.^[7]^ Currently in order to increase the survival time of advanced GC patients and to better control the local relapse, the importance of neoadjuvant treatments is being investigated. Many randomized controlled trials (RCTs) have been conducted and completed. However, the results from these RCTs were conflicting.^[8–10]^ Dutch Gastric Cancer Groups’ study demonstrated that neoadjuvant treatments cannot prolong survival rate.^[11]^ While, Xu's meta-analysis showed that neoadjuvant treatments potentially reduces overall mortality and contributes to lowering nodal stages.^[12]^

Pairwise meta-analysis cannot integrate all the information from different therapeutic methods in the mean time and we also cannot determine the superiority of a treatment. Network meta-analysis, a multiple-treatment comparison meta-analysis, combines the available information from pairwise comparisons of treatment A and treatment B and indirect comparisons C either a third intervention or a control condition. Thus, network meta-analysis can estimate the relative effectiveness among all interventions and rank the interventions even if head-to-head comparisons are lacking.^[13]^

The aim of our systematic review and network meta-analysis is to evaluate the relative efficacy of neoadjuvant treatments combined with surgery for GC in the improvement of 5-year survival rates, survival time, total and perioperative mortalities, R0 resection rate and postoperative complications using network meta-analysis.

## 2 Methods

### 2.1 Registration

We will perform our systematic review protocol according to the preferred reporting items for systematic review and meta-analysis protocols (PRISMA-P) extension statement.^[14]^ Our protocol has been registered on the international prospective register of systematic review (PROSPERO) network. The registration number was CRD42017074956.

### 2.2 Ethics and dissemination

#### 2.2.1 Ethics issues

This systematic review not need direct contact with the individual patients, and only included some previously published data for a further analysis. Therefore, this systematic review does not require ethics approval or obtaining informed consent.

#### 2.2.2 Publication plan

This systematic review will be published in a peer-reviewed journal and will be disseminated through conference posters or abstracts.

#### 2.2.3 Inclusion criteria

##### 2.2.3.1 Types of studies

Randomized or nonrandomized controlled trials (RCTs) will be included in this study. There were no limitations on year of publication, publication status;

##### 2.2.3.2 Types of participants

Patients with GC (diagnosed and classified as proposed by NCCN guideline ^[5]^) without age, gender, and racial limitations;

##### 2.2.3.3 Types of interventions

We will include the following 4 interventions: surgery alone (S), preoperative chemotherapy follow by surgery (CTS), preoperative radiotherapy follow by surgery (RTS), and preoperative chemoradiotherapy follow by surgery (CRTS);

##### 2.2.3.4 Type of outcomes

The primary outcomes are 5-year survival rates and overall survival. The secondary outcomes are total and perioperative mortalities, R0 resection rate, and postoperative complications.

##### 2.2.3.5 Information source

We will search 5 electronic databases as followings: PubMed, EMBASE, the Cochrane Central Register of Controlled Trials (CENTRAL), CNKI (Chinese National Knowledge Infrastructure), and CBM (Chinese Biological Medical Database). Also we will track the references of included articles and relevant systematic reviews and meta-analysis to identify other additional studies.

For conducting the retrieval, the following keywords were identified: gastric cancer, stomach neoplasms, gastric neoplasms, stomach cancer, surgery alone, preoperative chemotherapy, preoperative radiotherapy, preoperative chemoradiotherapy, and surgery.

##### 2.2.3.6 Data collection and analysis

###### 2.2.3.6.1 Data management

We will use ENDNOTE X7 (Thompson Reuters, CA) to manage literature search records. Before the literature selection, a pilot test will be conducted between the reviewers to ensure high inter-rater reliability.

###### 2.2.3.6.2 Selection process

According to formulated search strategy 2 reviewers will screen the title and abstract retrieved studies independently. If passed the title and abstract screening, the potentially eligible studies will be re-estimated by retrieving the full texts. And we also need a third reviewer in case of disagreement. According to PRISMA guidelines the study selection process will be illustrate in a flow diagram.^[15]^

###### 2.2.3.6.3 Data collection process

We will use Microsoft Excel 2010 (Microsoft Corp, Redmond, WA, www.microsoft.com) to extract data by create a standard data abstraction form. One reviewer will complete data extraction from all included studies. A second reviewer will check the consistency and accuracy of all extracted data. And disagreements will be resolved by a third reviewer. The extraction data items are as following: the first author, study design, year of publication, country, study period, sample size, age, sex, reference of study, regimen of neoadjuvant treatments performed, tumor type, and stage and outcomes of interest.

### 2.3 Quality of evidence assessment

According to Grading of Recommendations Assessment Development and Evaluation (GRADE), we will assess the quality of evidence as 4 levels following—high quality, moderate quality, low quality, and very low quality.^[16]^ Moreover, we will use the online guideline development tool (GDT, http://gdt.guidelinedevelopment.org/) to conduct this process.

### 2.4 Risk of bias individual studies

Cochrane Handbook version 5.1.0^[17]^ which assess 7 specific domains: sequence generation (selection bias), allocation concealment (selection bias), blinding of participants and personnel (performance bias and detection bias), incomplete outcome data (attrition bias), selective reporting (reporting bias), and other bias and the risk of bias of all included RCTs will be estimated using it. Based on criteria of the risk of bias judgment,^[18]^ we will evaluate methodological quality as low risk, high risk, or unclear risk of bias.

The risk of bias of included nonrandomized studies will be evaluated according to the tool for assessing risk of bias in nonrandomized studies of interventions (ROBINS-I),^[19]^ including bias due to confounding (preintervention), bias in selection of participants into the study (preintervention), bias in classification of interventions (at intervention), bias due to deviations from intended interventions (postintervention), bias due to missing data (postintervention), bias in measurement of outcomes (postintervention), bias in selection of the reported result (postintervention), and overall risk of bias. We will evaluate risk of bias as low, moderate, serious, critical risk of bias, and no information.

Two reviewers will complete the easement of risk of bias independently. The conflicts will be resolved by a third reviewer.

### 2.5 Geometry of the network

The function of *forest.netmeta* of R-3.4.1 (R Foundation for Statistical Computing, Vienna, Austria) will be used to draw network plots to describe and present the geometry of different interventions. And in order to represent different interventions nodes will be used and edges will be used to show the head-to-head comparisons among interventions.

### 2.6 Pairwise meta-analysis

Excel 2010 will be used to summarize and show data of all the included studies and their major characteristics related to the aim of this systematic review and meta-analysis. R-3.4.1 software will be used to conduct pairwise meta-analysis. The Higgins *I*^*2*^ statistic will be used to assess statistical heterogeneity among all included studies. If *I*^*2*^> 50% we considered *I*^*2*^ as large; medium if 25% < *I*^*2*^≥50%; and small if 0≤*I*^*2*^≥25%.^[20]^ If there is no evidence showed heterogeneity, we will perform fixed-effect model analysis; otherwise we will discuss the sources of heterogeneity using subgroup analysis or meta-regression. After excluding clinical heterogeneity, we will perform analysis using random-effect model. Dichotomous outcomes will be presented using the pooled odds ratios (ORs), with 95% confidence interval (95%CI). Mean difference (MD) with 95% CI will be presented for continue outcomes.

### 2.7 Network meta-analysis

We will use package *netmeta* version 0.9-8 of R-3.4.1 software to perform a network meta-analysis ^[21]^ to synthesize direct and indirect evidence for assessing the therapeutic effect among surgery alone, preoperative chemotherapy follow by surgery, preoperative radiotherapy follow by surgery, preoperative chemoradiotherapy follow by surgery. Node splitting method will be used to assess inconsistency between direct and indirect comparisons if a loop connecting 3 arms existed. The treatment ranking will present by *P*-scores based on the point estimates and standard errors of the network assesses.

### 2.8 Other analyses

#### 2.8.1 Subgroup and sensitivity analyses

Considered of possible significant heterogeneity or inconsistency, we will use subgroup analysis to find the possible sources. Subgroup analyses are designed for age, sex, and different preoperative chemotherapy methods.

We also will assess the sensitivity of results by analyzing studies including patients without complications, the studies without missing data.

#### 2.8.2 Publication bias

We will use STATA V.12.0 software (Stata Corporation, College Station, Texas) to draw a comparison-adjusted funnel plot to identify whether there will be a small sample effect among the networks.

## Authors’ contributions

LB, JZY, LQ, and ZH planed and designed the research; YZY, JZY, DHR and WZJ tested the feasibility of the study; LB wrote the manuscript; all authors approved the final version of the manuscript.
